# Comparative assessment of anatomical details of thoracic limb bones of a horse to that of models produced via scanning and 3D printing

**DOI:** 10.1186/s41205-019-0050-2

**Published:** 2019-08-02

**Authors:** Daniela de Alcântara Leite dos Reis, Beatriz Laura Rojas Gouveia, José Carlos Rosa Júnior, Antônio Chaves de Assis Neto

**Affiliations:** Department of Surgery, School of Veterinary Medicine and Animal Science, University of São Paulo, Av. Prof. Dr. Orlando Marques, 77, ZC, São Paulo, SP 05508-270 Brazil

**Keywords:** Additive manufacturing, 3D printing, Anatomical models, Comparative anatomy

## Abstract

**Background:**

Three-dimensional (3D) scanning and printing for the production of models is an innovative tool that can be used in veterinary anatomy practical classes. Ease of access to this teaching material can be an important aspect of learning the anatomy of domestic animals. In this study, a scanner was used to capture 3D images and a 3D printer that performs die-cast printing was used to produce skeletal models of the thoracic limb of a horse.

**Methods:**

Bones from a horse were selected for scanning and creation of 3D-printed models. The printer used a filamentous thermoplastic material (acrylonitrile-butadiene-styrene [ABS]) which was deposited together with a support resin. Comparisons of the anatomical characteristics (measurements from the original and printed bone) were analyzed to determine the *p*-value.

**Results:**

Bones from the thoracic limb: scapula, humerus, radius and ulna, carpus and phalanges were used to produce digital and physical models for 3D impressions. Then the anatomical characteristics of the 3D printed models were compared with those of the original bones. The *p*-value was measured to be 0.9126, indicative of a strong evidence of similarity between the 3D-printed models and specimens. Thus, there was no significant statistical difference between the models and the original anatomical parts.

**Conclusions:**

The anatomical characteristics were successfully identified in the 3D-printed copies, demonstrating that models of animal bones can be reproduced using 3D printing technology for use in veterinary education.

## Background

Several obstacles hinder the easy acquisition and preparation of anatomical specimens. The high cost of preserving cadavers and acquiring equipment needed to maintain them have led many laboratories to abandon these practices. Many institutions and universities primarily rely on the use of books with two-dimensional images and written information, as well as classes, through which information about clinical cases are passed verbally; photographs are used for teaching anatomy, given the lack of feasibility of using corpses for such studies. However, researchers have shown that such methods are more effective for learning when combined with other methods, such as the use of anatomical models [1–4].

Although the theoretical teaching of anatomy is extremely important, practical studies are essential to consolidate the theoretical aspects. For practical teaching to be successful, it is important that the anatomical specimens being used are in good condition; the color, texture, flexibility, and other characteristics accurately represent those found in a living animal. Technological resources can contribute to the teaching of veterinary anatomy, and make this discipline more interesting and accurate.

Colored human skull models were produced by 3D printing technology and were advantageous in assisting anatomical study, especially in structure recognition, compared with traditional educational cadaveric material [5]. The scanning of anatomical parts and the printing of replicas using a 3D printer can be a very efficient resource in the production of didactic material [6].

The use of 3D scanning and printing for the production of models is an innovative tool that can be used to make osseous models for use in veterinary anatomy practical classes [5, 7, 8], planning and reconstructive surgery [9–16], and prototyping of cranioplasty [17, 18]. The anatomical 3D model has been gaining strength globally, since they are becoming increasingly fast, economical, and easy-to-use techniques [19].

The aim of this study was to produce skeletal models of the thoracic limb of a horse; thus, proposing to make available the models produced as a study tool in practical classes of veterinary anatomy.

## Methods

### Specimens

The bones of an adult horse used in this study were from the Veterinary Anatomy Laboratory of the School of Veterinary Medicine and Animal Science (FMVZ) of the University of São Paulo (USP). Bones of the left thoracic limb selected for scanning and creation of 3D models are as follows: the scapula, humerus, radius and ulna, metacarpal bones, accessory carpal bone, ulnar carpal bone, intermediate carpal bone, radial carpal bone, second carpal bone, third carpal bone, fourth carpal bone, proximal sesamoids, proximal phalanx, middle phalanx, distal phalanx, and distal sesamoid.

### Scanner

The skeletal parts were digitalized using the “Go!SCAN 3D” model Creaform® (Lévis, Quebec, Canada). This scanner has two high-definition digital cameras, each surrounded by a set of four white LED (Light Emitting Diode) bulbs, and a projector that emits a white light pattern. The cameras detect the surface of an object and acquire images that are displayed by the software VXelements (Lévis, Quebec, Canada) in the form of a mesh composed of thousands or even millions of triangles. This software handles the acquisition and processing of 3D data generated from 3D scanner digitalization. The bone images were formed in real-time, and a file with the extension “.csf” was created automatically. The digitalized images were edited using the software Geomagic (Cary, NC, USA). This software allows the correction of the generated images, using tools to exclude some uneven surfaces, flatten bumps, smoothen meshes, reduce noise, and fill flaws.

The scanner was calibrated before image acquisition to adjust the total size of the scanned objects. The scanner was operated manually but the system automatically positions the point clouds. For the automatic assembly of the point clouds was used points reflective targets on the surface of bones. These targets are illuminated by LEDs (light emitters) arranged around the cameras of the scanner to get cloud points. By registering these targets, the system calculates the position of the scanner and allows it to be moved in relation to the scanned object, which enables the object surface to be scanned without having to generate multiple files for different viewpoints of the part. The distance from the scanner to the bones (approximately 30 cm) was controlled using a scale bar on the software screen of the scanner, VXelements. The 3D point cloud was then transferred to the Geomagic® editing software to undergo an editing process that allows image enhancement. The software is also an interface software with the ability to convert the 3D file into print layers.

### 3D printing

The digital files used to produce the parts (biomodels) were printed using the 3D printer - model Mojo® (Rehovot, Israel). The printer uses a thermoplastic material in the form of a filament (acrylonitrile-butadiene-styrene, ABS) that is deposited together with a support resin. It takes a few hours to print, with the time varying depending on the size and the details of the anatomical part. Printed parts are cleaned in a WaveWash 55 - Stratasys® washer to remove the support resin, thereby leaving only the part that was being manufactured. In this cleaning process, which lasts approximately 8 h, the washer uses a specific cleaning agent (Ecoworks Tablets Cleaning Agent®) that removes only the support resin. The printed models were infilled with the thermoplastic material.

The printing time was recorded. Certain models were not printed to real scale because of size limitations of the print tray.

### Comparative analyses of the bones and models

Specific pertinent anatomical details were identified/selected for each bone used in this study. The dimensions of these structures in the original bones and in the 3D printed models were measured by a single observer using a digital caliper. The anatomical characteristics of the bone parts and the 3D printed models were analyzed visually and compared with details in current veterinary anatomy textbooks of domestic mammals [20–22]. The weights of the bones and the models were each measured using a digital scale and the results compared. Some anatomical characteristics that were present in the bones and in the 3D printed models were measured such as: scapula (width, neck, and glenoid cavity of the scapula), humerus (trochlea humeri, deltoid tuberosity, intermediate tubercle, greater tubercle, lesser tubercle, intertubercular groove, and head of the humerus), radius (radial tuberosity and processus styloideus medialis) and ulna (thickness of the processus anconeus and tuber olecrani), carpal bone (accessory carpal, ulnar carpal bone, intermediate carpal bone, radial carpal bone, second carpal bone, third carpal bone, and fourth carpal bone), metacarpal bones II-III-IV, phalanx (proximal, middle, and distal), and sesamoids (proximal and distal). Each of these measurements was performed three times by a single observer using a digital caliper.

### Statistical analysis of measurements

To validate the quality of the 3D printed models and to ensure that there was no loss of scale and size when compared to the real bones, the t-teste was applied to compare the general mean of dimensions from the 3D printed models against the general mean of dimensions from real bones.

## Results

### Printing time and consumption of materials

**Fig. 1:**
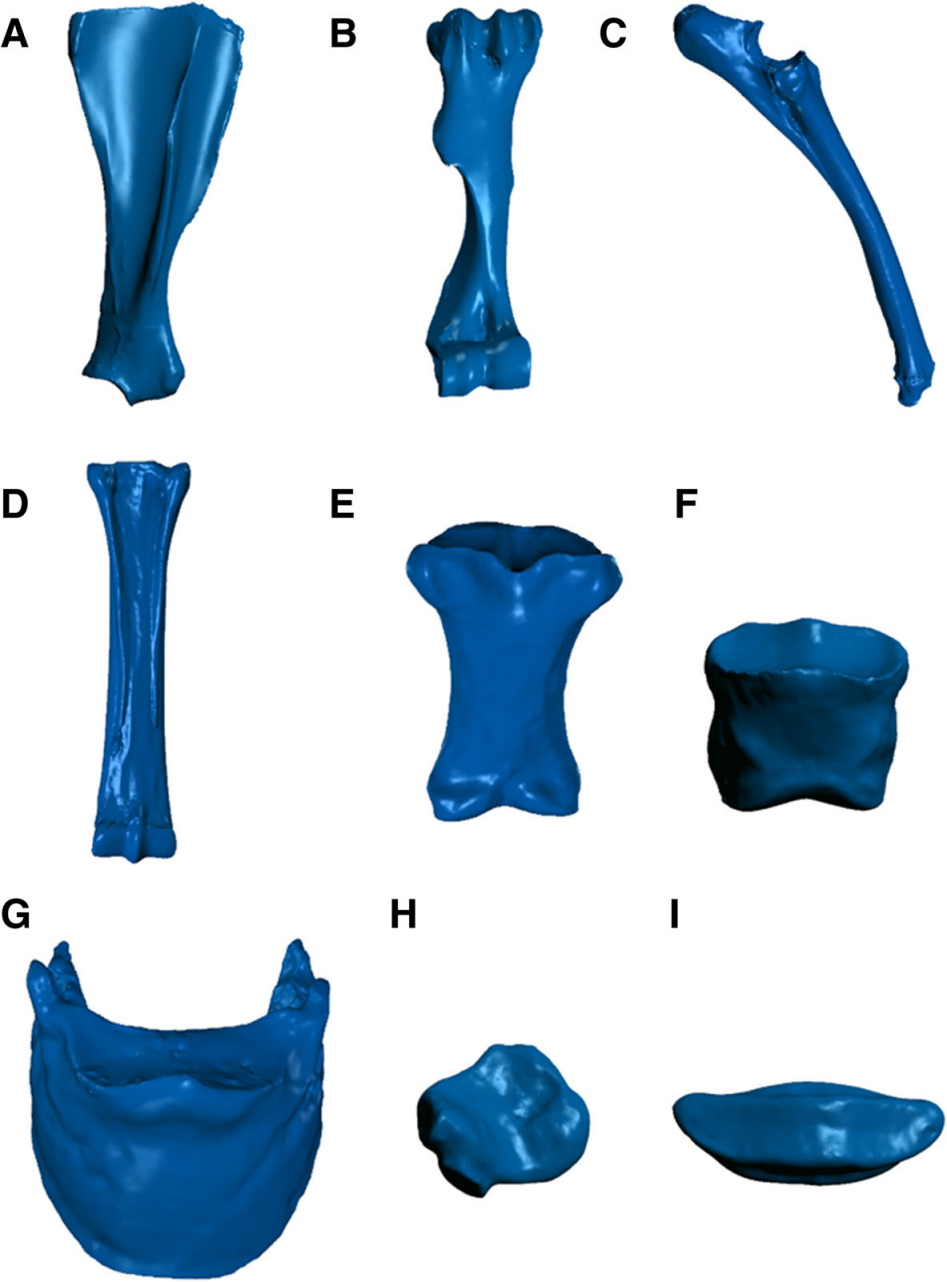
STL (Standard Triangle Language) image produced by the “Geomagic” software (**a**) scapula, ventral view; (**b**) humerus, cranial view; (**c**) radius and ulna, lateral views; (**d**) os metacarpale, palmar view; (**e**) phalanx proximalis, palmar view; (**f**) phalanx media, dorsal view; (**g**) phalanx distalis, dorsal view; (**h**) os carpi accessorium; (**e**) sesamoideum distale, dorsal view

The printing time varied with the size and complexity of the part; the rate of consumption of materials used in the printing varied as well. The bone that took the most time to print was the humerus, which took approximately 14 h. Consumption of ABS and support material also depended on the size of the bone and on how the digital model was positioned in the print tray before the process started. The bone that consumed the most material during printing was the scapula (43.8 cm^3^) (Table 1) (Fig. 1).

**Table 1:** Print time of 3D-printed models and consumption of acrylonitrile-butadiene-styrene and support material

Bones	Print time (hours)	ABS material (cm^3^)	Support material (cm^3^)
Scapula	10 h31 min	52.8	48.3
Humerus	14h16min	87.2	43.0
Radius/Ulna	10 h	51.8	28.3
Metacarpal	9h42min	62.3	28.5
Accessory carpal	1h51min	8.7	4.5
Intermediate carpal	1h43min	8.2	3.6
Radial carpal	2h23min	10.4	5.6
Ulnar carpal	1h10min	5.2	2.4
Fourth carpal	1h6min	4.9	1.9
Second carpal	32 min	2.7	0.6
Third carpal	1h56min	9.6	3.4
Proximal phalanx	3h51min	31.8	7.3
Middle phalanx	3h5min	18.3	7.5
Distal phalanx	5h46min	26.5	16.5
Proximal sesamoid 1	1h5min	4.7	2.1
Proximal sesamoid 2	53 min	4.3	1.5
Distal sesamoid	1h7min	3.8	2.6

*ABS* acrylonitrile-butadiene-styrene

### Comparison of weights between the bones and 3D-printed models

The thermoplastic material used for 3D printing allowed us to manufacture lightweight models with specific rigidity and resistance. All-natural bones weighed much more that their representative models. For example, the actual horse’s humerus weighed 763 g while its model weighed 148 g.

### Anatomical characteristics of the 3D printed model

**Fig. 2:**
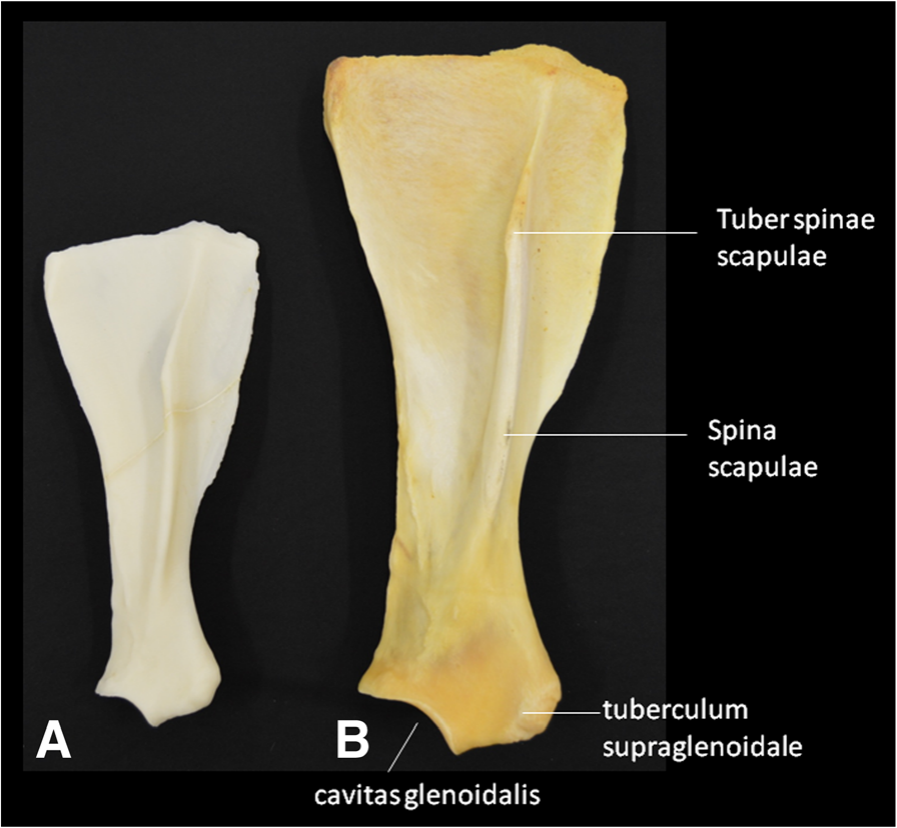
Scapula, lateral view. **a** Model printed at 80% of the original size and **b** original bone

**Fig. 3:**
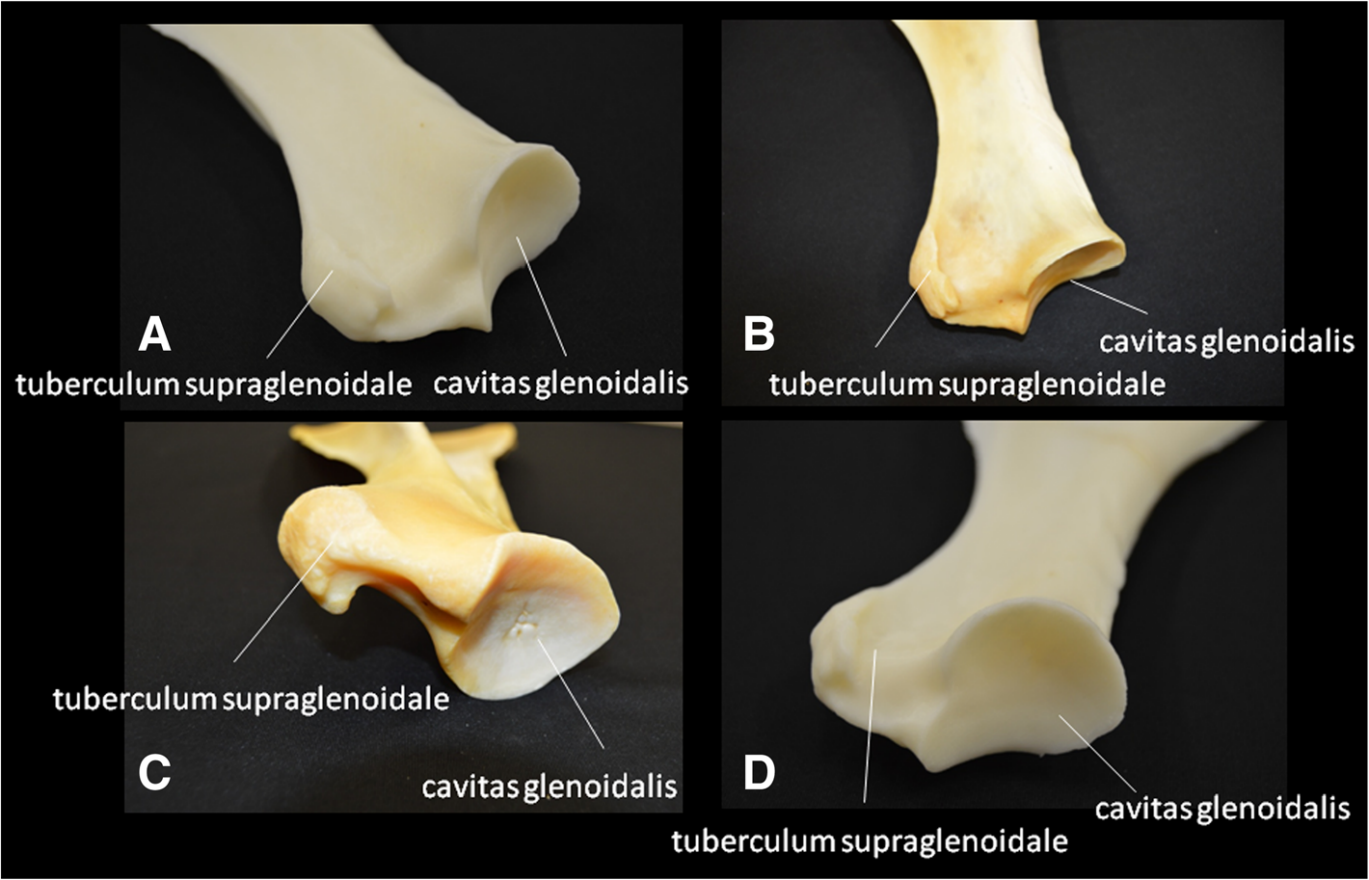
Scapula, where (**b**) and (**c**) are the original bones; (**a**) and (**d**) are models. Note the cavitas glenoidalis and tuberculum supraglenoidale

The 3D models were printed from the interface that is able to convert the 3D file into print layers. When performing the visual analysis of the scapula and its printed copy, there was a significant difference in relation to the length and the width, because limitations of the printer meant the copy was produced with 75% of the original size. Anatomical features such as the spine of the scapula, the tubercle of the spine of the scapula, and the glenoid cavity were well outlined in the printed model. The model of the scapula was produced in two parts, followed by the union of these parts with instant glue (Figs. 2 and 3) (Table 2).

**Table 2:** Measurements of the anatomical characteristics of the natural and 3D-printed model of the scapula

Anatomical characteristics	Bones (mm)	3D-Printed (mm)	Corrected value
Width of scapula (measured between cranial angle and caudal angle)	177.3	120.4	160.5
Neck of scapula	67.4	46.2	61.6
Glenoid cavity - craniocaudal measurement	60.6	42.3	56.4
Glenoid cavity - measured lateromedially	52.7	35.3	47.1
Tuberosity of spine of scapula (thickness)	8.3	5.1	6.8
Supraglenoid tubercle - measured lateromedially	28.6	19.9	26.5
Length of the scapula (from the cranial margin of the glenoid cavity to dorsal border)	410.3	278.9	371.9

**Fig. 4:**
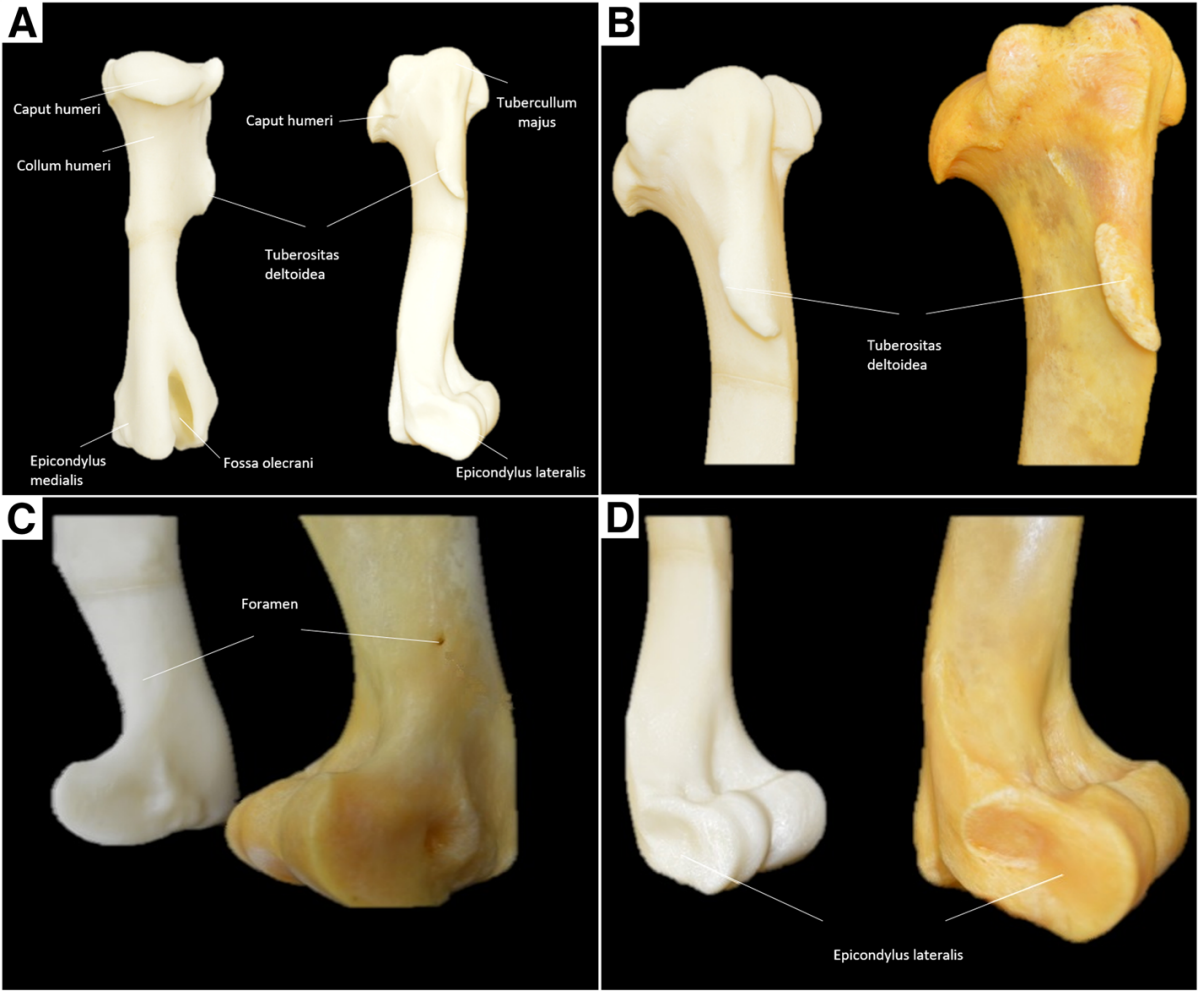
3D-printed horse humerus. **a** Caudal and lateral views, **b** Lateral view of the proximal horse humerus; 3D-printed bone (left) and **b** original bone (right). **c** Lateral views of the distal humeris; 3D-printed bone (left) and original bone (right). The details of the foramen and epicondylis lateralis are clearly visualized. **d** Medial views of the distal humeris; 3D-printed bone (left) and original bone (right). The details of the epicondylis medialis are clearly visualized

The humeral model was different in relation to the size of the bone, since it was printed at 80% of the original size. There were no differences in the anatomical specificities of the original bone. Anatomical features such as the deltoid tuberosity, greater tubercle, lesser tubercle and intermediate tubercle, humeral head and neck, intertubercular grooves, trochlea and olecranon fossa were well outlined in the printed model. The foramen nutricium was not visible in the printed model (Fig. 4) (Table 3).

**Table 3:** Measurements of the anatomical characteristics of the natural and 3D-printed model of the humerus

Anatomical characteristics	Bones (mm)	3D-Printed (mm)	Corrected value
Trochlea of the humerus -measured lateromedially	74.1	58.5	73.1
Deltoid tuberosity – thickness	12.2	9.4	11.7
Intermediate tubercle **–** lateromedial thickness	21.3	16.7	20.9
Greater tubercle	21.4	16.8	21.0
Lesser tubercle	16.7	11.7	14.6
Intertubercular groove – between the intermediate tubercle and greater tubercle	22.8	17.9	22.4
Intertubercular groove **–** between the intermediate tubercle and lesser tubercle	18.3	15.0	18.7
Head of the humerus - measured lateromedially	67.4	50.4	63.0

**Fig. 5:**
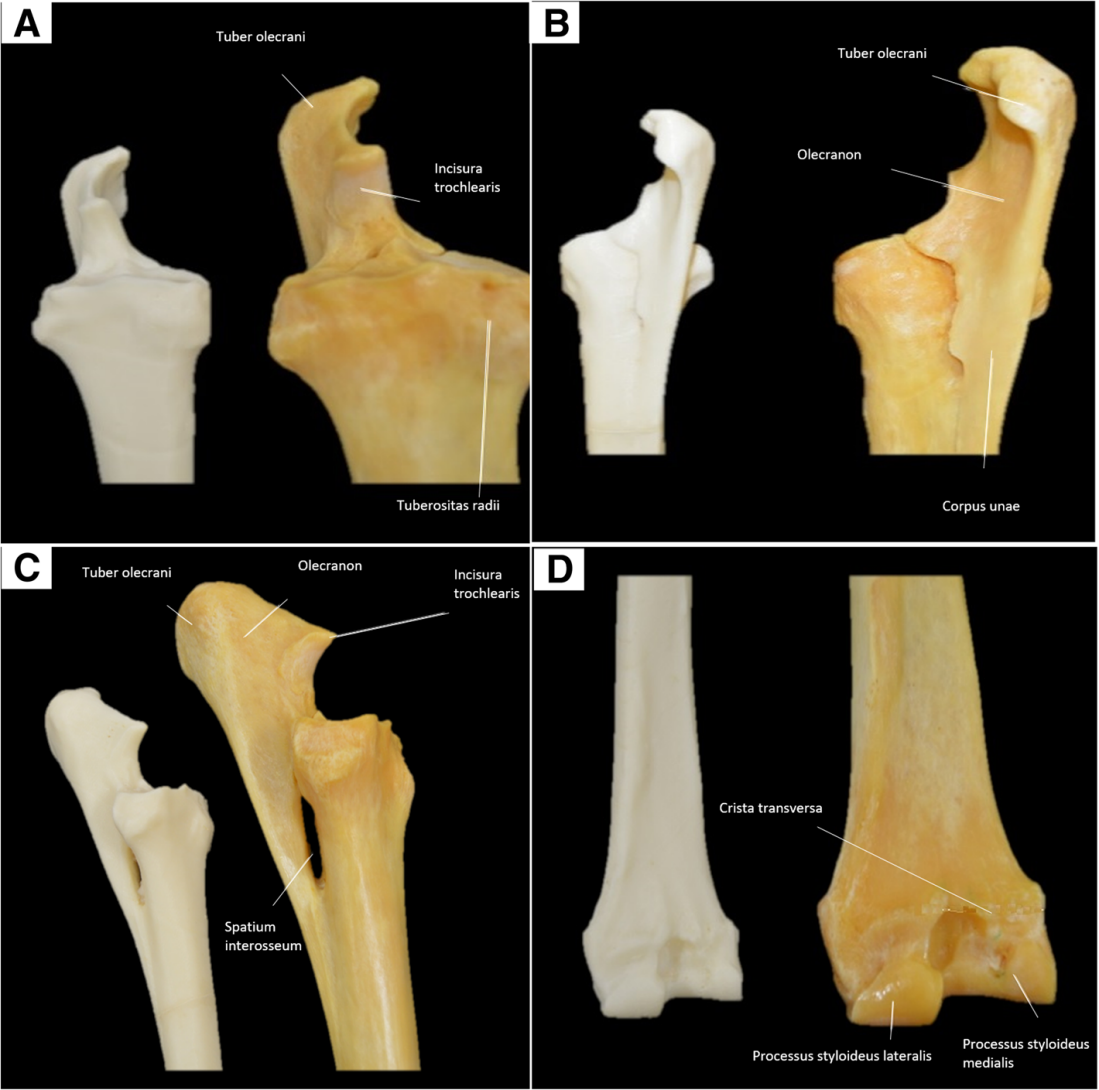
**a** Matched pairs of 3D-printed (left) and original (right) proximal radius and ulna bones. **b** Caudomedial view of the 3D-printed (left) and original bone (right). **c** Lateral view of the 3D-printed (left) and original bone (right). **d** Caudal view of the distal radius bone of a horse; 3D-printed bone (left) and original bone (right)

The 3D printed model of the radius and ulna were printed at 80% of the size of the original bones. Anatomical features such as the anconeal process, trochlear notch, olecranon tuberosity, and the styloid process were visually identified in the model. Details such as the interosseous space were also observed (Fig. 5) (Table 4).

**Table 4:** Measurements of the anatomical characteristics of the natural and 3D-printed model of the radius/ulna

Anatomical characteristics	Bones (mm)	3D-Printed (mm)	Corrected value
Radial tuberosity	81.1	56.0	80.0
Processus anconeus – measured lateromedially	15.9	11.1	15.7
Tuber olecrani *–* measured lateromedially	37.9	25.4	36.3
Processus styloideus *medialis* - measured lateromedially	27.0	18.8	26.9

**Fig. 6:**
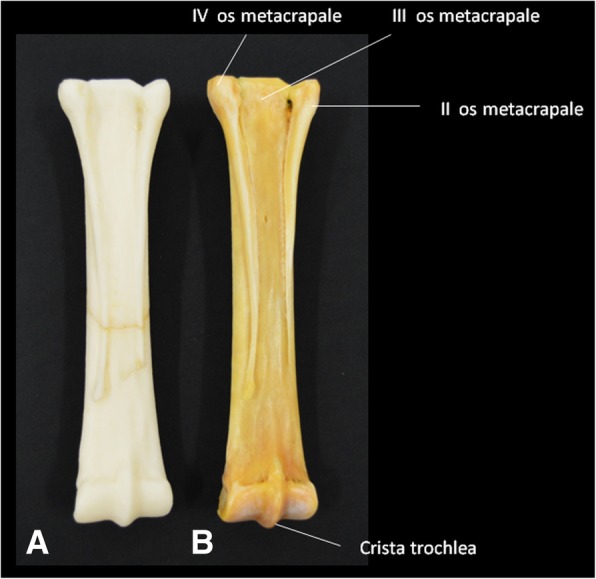
Palmar views of the horse metacarpal bone. **a** 3D-printed bone, and **b** original bone

**Fig. 7:**
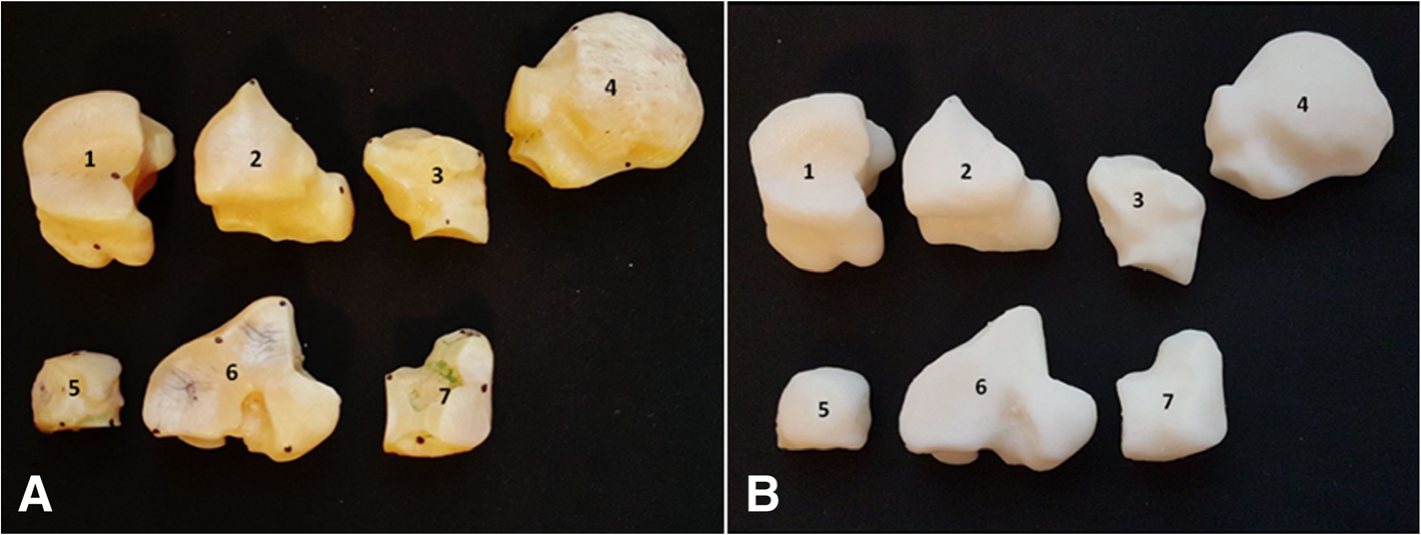
**a** Bones of the carpus and **b** 3D printed models. 1- carpi radiale, 2- carpi intermedium, 3- carpi ulnare, 4- carpi accessorium, 5- carpale II, 6- carpale III, 7- carpale IV

**Fig. 8:**
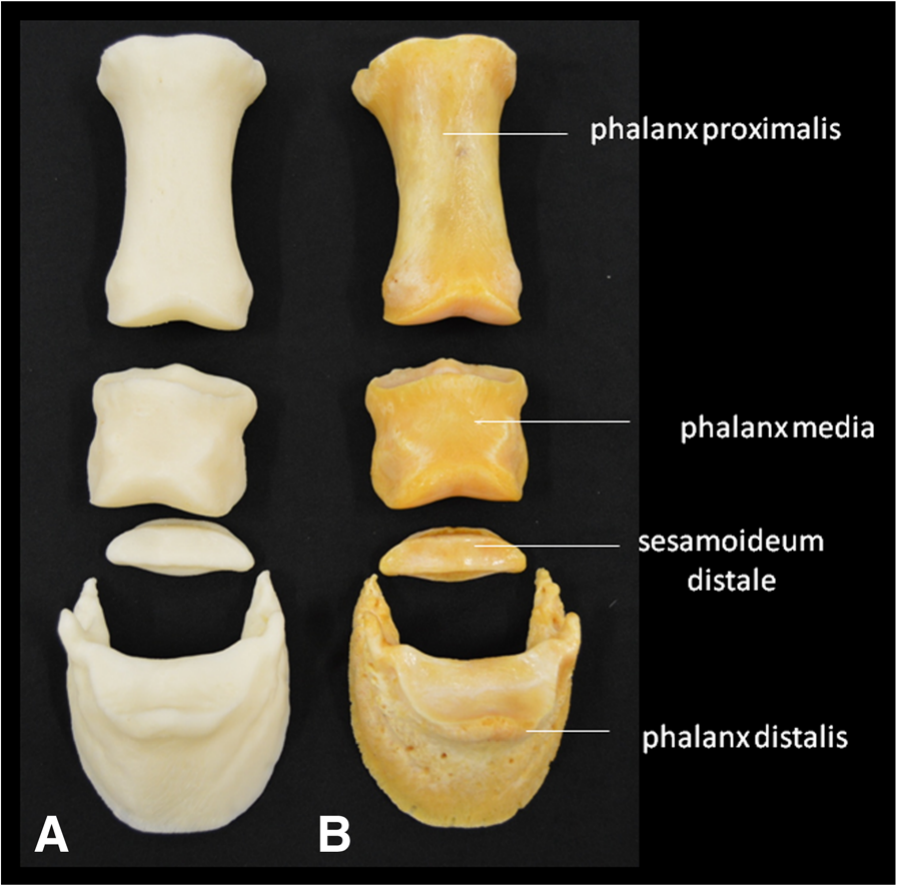
Dorsal view of the proximal, middle, and distal phalanges and distal sesamoid bones of the horse. Here (**a**) 3D model and (**b**) original bones

Printed copies of the carpal bones (Table 5), metacarpal bones II, III and IV (Table 6), and phalanges (Table 7) were done on a real scale. Anatomical details such as the trochlear crest as well as the distal end of the metacarpal II were visually identified. The foramen nutricium has not been identified in the printed specimen. However, a small hole can be made to represent it after printing. The distal end of the metacarpal IV was not present in the bone, and therefore was not shown in the printed copy. Although they were produced in real scale, the measurements made in the anatomical details of the model presented discreetly larger values, due to the finishing of the edges of the parts, which are slightly more rounded. The foramina of the distal phalanges were not observed in the model; however, a small hole could be made to represent it after the printing (Figs. 6, 7, and 8). Finally, means of dimensions were compared by t-test (*p* = 0,9126), indicating a consistent evidence of similarity between 3D printed and real bones.

**Table 5:** Measurements of the anatomical characteristics of the natural and 3D-printed models of the carpal bones

	Bones (mm)	3D-Printed model (mm)
Radial carpal bone		
x axis	24.2	25.1
y axis	38.1	39.0
Intermediate carpal bone		
x axis	33.9	34.0
y axis	38.5	39.5
Ulnar carpal bone		
x axis	29.9	30.9
y axis	30.4	31.2
Accessory carpal bone		
x axis	48.2	48.1
y axis	37.8	38.8
Second (II) Carpal bone		
x axis	23.2	24.2
y axis	20.5	20.6
Third (III) Carpal bone		
x axis	43.8	44.5
y axis	33.7	34.7
Fourth (IV) Carpal bone		
x axis	25.2	26.1
y axis	30.3	30.6
Distal Sesamoid		
x axis	48.2	48.5
y axis	17.7	18.6

**Table 6:** Measurements of the anatomical characteristics of the natural and 3D printed models of the metacarpal bones II-III-IV

Anatomical characteristics	Bones (mm)	3D-printed models (mm)
Thickness of the Trochlea of the metacarpal bone III - measured lateromedially	50.1	50.5
Trochlea crest of the metacarpal bone III	13.5	13.7
Thickness of the metacarpal bone II - craniocaudal measurement	21.9	23.2
Thickness of the metacarpal bone IV – craniocaudal measurement	20.3	20.4
Thickness of the distal end (button) of the metacarpal bone II – measured lateromedially	5.5	5.8
Length of metacarpal bone III – from the articular surface to crest of the trochlea	248.6	248.9

**Table 7:** Measurements of the anatomical characteristics of the natural and 3D-printed models of the phalanges

Anatomical characteristics	Bones (mm)	3D-Printed model (mm)
Length of proximal phalanx	84.2	84.6
Thickness of the proximal articular surface (between eminences) – measured lateromedially	56.0	56.5
Distal articular surface thickness (between eminences) – measured lateromedially	47.7	47.9
Length of middle phalanx	44.8	45.1
Proximal articular surface width	53.4	53.6
Superficial caudal articular width	51.4	52.2
Distal phalanx		
Distance from the extensor process to sole margin	58.3	59.8
Distance between lateral and medial palm processes	67.5	67.8
Lateral palmar process thickness	5.0	5.3
Medial palmar process thickness	4.5	4.7

## Discussion

This research describes an important method for producing accurate 3D horse skeletal models (replicas) using a manual scanner and a 3D printer. A comparative analysis of the anatomical details between the replicas and the bones was carried out to verify if the visual features of the replicas were similar to the original bones for future use as specimens for anatomical studies. Much of the original anatomical features of the scapula, humerus, radius and ulna bones, carpal bones, metacarpals, and phalanges were readily identified. These results are consistent with those previously reported by other authors [23].

Comparisons of the measurements of some anatomical structures performed on the bones and on the copies did not show any significant difference, suggesting that 3D models can accurately display most anatomical characteristics of the original bone. As such, they are useful in the study of veterinary anatomy [4, 24].

The results showed that the differences between the measurements of the anatomical structures of the bones and their replicas were small, so the osteometric analysis we performed revealed that there was no significant difference in the shape and dimensions of the printed models when compared to the real bones. Similar results were obtained in studies that evaluated the accuracy of the 3D scanning system [23, 25]. The main differences were observed in the larger models such as the scapula, humerus, radius, and ulna. Replicas of smaller bones such as carpal bones showed discrete differences in dimensions because their margins were rounder; however, these deviations did not lead to any significant representational errors between the models produced and the original parts.

In the present study, the foramen nutricium was not successfully visualized in the 3D-printed models. Le at al. [23] reported that details could be reproduced through editing software and demonstrated on digital models. Bone replicas that are larger than the print tray may have several separate parts printed and then joined together without compromising the visual appearance of the anatomical structures [25].

The effectiveness of learning with the 3D-printed models will be analyzed in another study, which will assess the performance of students in a classroom using the printed models and real bones, over 2 years, based on practical exams. Students can be objectively evaluated by directing them to identify structures in the cadaver specimens and compare them with the printed material.

The costs involved in manufacturing the parts (ABS filament and support material) via 3D printing are still smaller when compared to the costs for producing anatomical parts through techniques performed in anatomy laboratories or the cost of buying plastic parts [6]. Moreover, the manufactured models are sufficiently detailed in their anatomy to constitute an alternative teaching material.

There are plastic models of anatomical parts in the market that are often used in some educational institutions. These models are copies or molds produced on a large scale based on “hypothetical” or “caricature” anatomical specimens and they often lack specific and important anatomical details. Although they may be suitable for some teaching programs that have lower academic requirements, they are not ideal for teaching anatomy at the academic level expected of students studying veterinary medicine [6].

After investing in scanning and printing equipment, the cost for model production is relatively cheap [23]. In this study, the most expensive printed bone was the humerus and it cost approximately US$ 70.45, but this can further be lowered. This cost is due to the printer model used in the study, which operates using high-cost materials. However, we believe that the 3D printing cost becomes cheaper every year. Additionally, by scanning the parts, digital files are created, and these can be printed in companies that offer 3D printing service, which may be a useful alternative in situations when the institution does not own a 3D printer.

The time needed to produce these models is an advantage when compared with the time needed to obtain bones and prepare them for student use in the laboratory. However, with creating bone models, there is a short initial scanning time with scans of the bones lasting around 50 min. The printing time might reduce as the professional handling the scanner develops skill and familiarity with the device. Subsequent models can be printed relatively quickly, where large bones such as the humerus take approximately 14 h to print, but small bones such as the carpals take 2 hrs.

Because the models are produced from a thermoplastic material which weighs much less than the bones, their use as teaching material is not restricted only to the models being used in anatomy laboratories, they can also be used in other places, such as libraries and classrooms. The heaviest replica we produced was the horse humerus, which weighed 148 g. The models averaged about one fifth of the weight of natural bones.

3D scanning and printing can also be used to reproduce the bones of rare or endangered species that are often inaccessible for educational purposes. Copies of the skeletons of these animals can be manufactured for use without fear of damage. In case of damage, universities can print a new part and thus enable the study of the anatomy of these animals [25].

This technology may also be useful in research for manufacturing anatomical models for the discussion of new surgical strategies. Another study tool that can be quite useful is the database of the images that were scanned. These images may be interactively applied in classrooms or employed as a source of material for creating websites and smartphone applications aimed at teaching anatomy.

## Conclusions

This study introduces an innovative and high-level project of technological development aimed at creating a method for the fast production of 3D printed models of bones using 3D scanning and 3D printing technologies. The accuracy and reliability of the printed models was confirmed by visual analysis of their anatomical characteristics, measurements of their structures, and comparisons with the bones. Thus, through future studies involving tests of 3D-printed models with students in veterinary anatomy classes, these models may soon be found to be an aid in anatomy lessons and may serve as reliable alternatives in the study of veterinary anatomy.

## Data Availability

All data generated during this study are available from the corresponding author.
